# 1-(2-Chloro-5-nitro­phen­yl)-3-(2,2-di­methyl­propion­yl)thio­urea

**DOI:** 10.1107/S1600536809024672

**Published:** 2009-07-01

**Authors:** Aamer Saeed, Rasheed Ahmad Khera, Jim Simpson, Roderick G. Stanley

**Affiliations:** a Department of Chemistry, Quaid-i-Azam University, Islamabad 45320, Pakistan; b Department of Chemistry, University of Otago, PO Box 56, Dunedin, New Zealand

## Abstract

With the exception of the C atoms of two of the methyl groups of the *tert*-butyl substituent, all of the non-H atoms of the title compound, C_12_H_14_ClN_3_O_3_S, lie on a mirror plane. The 2-chloro-5-nitro­phenyl and 2,2-dimethyl­propionyl substituents are, respectively, *cis* and *trans* relative to the thio­carbonyl S atom across the two C—N bonds. Intra­molecular N—H⋯O and C—H⋯S hydrogen bonds form *S*(6) ring motifs, also in the mirror plane. Despite the presence of two N—H subsituents, no inter­molecular hydrogen bonds are observed in the crystal structure. Weak π–π contacts [centroid–centroid distances of 4.2903 (17) Å] involving adjacent aromatic rings link the mol­ecules in a head-to-tail fashion above and below the mol­ecular plane.

## Related literature

For the use of thio­urea derivatives in organic synthesis, see: Eynde & Watte (2003 ▶); Fu *et al.* (1999 ▶); Rashdan *et al.* (2006 ▶); Maryanoff *et al.* (1986 ▶); Wang *et al.* (2005 ▶); Saeed *et al.* (2008 ▶); and in analysis, see: Koch (2001 ▶). For their bioactivity and pharmaceutical applications, see: Upadhyaya & Srivastava (1982 ▶); Krishnamurthy *et al.* (1999 ▶); Blum & Hayes (1979 ▶); DeBeer *et al.* (1936 ▶). For related structures, see: Saeed & Flörke (2007*a* ▶,*b* ▶); Yusof *et al.* (2006 ▶, 2008 ▶). For reference structural data, see: Allen *et al.* (1987 ▶).
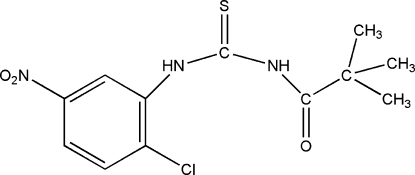


## Experimental

### 

#### Crystal data

C_12_H_14_ClN_3_O_3_S
*M* *_r_* = 315.77
Orthorhombic, 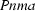

*a* = 9.529 (2) Å
*b* = 6.546 (2) Å
*c* = 22.166 (6) Å
*V* = 1382.7 (7) Å^3^
*Z* = 4
Mo *K*α radiation
μ = 0.44 mm^−1^
*T* = 89 K
0.36 × 0.09 × 0.06 mm

#### Data collection

Bruker APEXII CCD area-detector diffractometer
Absorption correction: multi-scan (*SADABS*; Bruker, 2006 ▶) *T*_min_ = 0.833, *T*_max_ = 0.974
12525 measured reflections
1434 independent reflections
1166 reflections with *I* > 2σ(*I*)
*R*_int_ = 0.069

#### Refinement

*R*[*F* ^2^ > 2σ(*F* ^2^)] = 0.033
*wR*(*F* ^2^) = 0.092
*S* = 1.09
1434 reflections
130 parameters
H atoms treated by a mixture of independent and constrained refinement
Δρ_max_ = 0.27 e Å^−3^
Δρ_min_ = −0.43 e Å^−3^

### 

Data collection: *APEX2* (Bruker, 2006 ▶); cell refinement: *APEX2* and *SAINT* (Bruker, 2006 ▶); data reduction: *SAINT*; program(s) used to solve structure: *SHELXS97* (Sheldrick, 2008 ▶); program(s) used to refine structure: *SHELXL97* (Sheldrick, 2008 ▶) and *TITAN2000* (Hunter & Simpson, 1999 ▶); molecular graphics: *SHELXTL* (Sheldrick, 2008 ▶) and *Mercury* (Macrae *et al.*, 2006 ▶); software used to prepare material for publication: *SHELXL97*, *enCIFer* (Allen *et al.*, 2004 ▶), *PLATON* (Spek, 2009 ▶) and *publCIF* (Westrip, 2009 ▶).

## Supplementary Material

Crystal structure: contains datablocks global, I. DOI: 10.1107/S1600536809024672/lh2851sup1.cif

Structure factors: contains datablocks I. DOI: 10.1107/S1600536809024672/lh2851Isup2.hkl

Additional supplementary materials:  crystallographic information; 3D view; checkCIF report

## Figures and Tables

**Table 1:** Hydrogen-bond geometry (Å, °)

*D*—H⋯*A*	*D*—H	H⋯*A*	*D*⋯*A*	*D*—H⋯*A*
C9—H9⋯S1	0.95	2.51	3.197 (3)	130
N2—H2*N*⋯O1	0.97 (3)	1.82 (3)	2.653 (3)	141 (3)

**Fig. 1.**
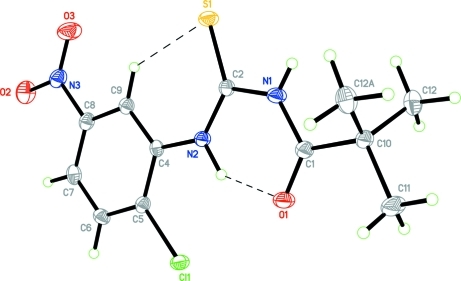
The structure of (I) with displacement ellipsoids for the non-hydrogen atoms drawn at the 50% probability level. Intramolecular hydrogen bonds are drawn as dashed lines. The atom labeled C12A is related to the C12 atom by the symmetry operation x, -y + 1/2, z.

**Fig. 2.**
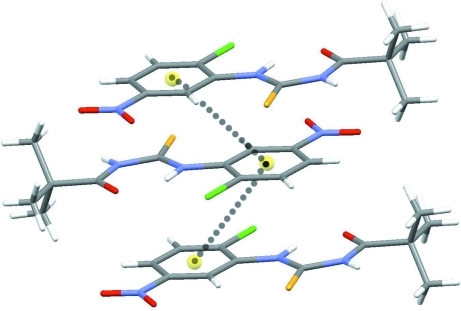
π–π interactions for (1).

**Fig. 3.**
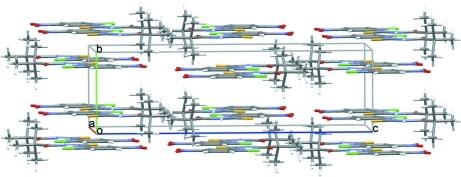
Crystal packing for (I) viewed down the a axis.

## supplementary crystallographic information

### Comment

Substituted thioureas are versatile building blocks for the synthesis of a variety of heterocyclic compounds with a broad range of useful applications and exhibiting a wide range of bioactivity. Solid-phase Biginelli pyrimidine synthesis (Eynde & Watte, 2003) and synthesis of imidazoline derivatives (Fu *et al.*, 1999) have been carried out using resin-bound thioureas. Pyridyl thioureas are switchable anion receptors (Rashdan *et al.*, 2006); thioureas are also efficient guanylating agents (Maryanoff *et al.*, 1986) while *N*,*N*-dialkyl-N-aroyl thioureas are efficient ligands for the separation of platinum group metals (Koch, 2001). Acyl thioureas are well known for their superior pesticidal, fungicidal, antiviral and plant-growth regulatory activities (Upadhyaya & Srivastava, 1982). 1,3-Dialkyl or diaryl thioureas show powerful antifungal activity against plant pathogens Pyricularia oryzae and Drechslera oryzae (Krishnamurthy *et al.*, 1999). Substituted thioureas are potent enhancers of 30S dynein ATPase activity and inhibitors of 14S dynein ATPase activity (Blum & Hayes, 1979). Aryl and alkyl-aryl thioureas display strong hypnotic potency in mice (DeBeer *et al.*, 1936). 1-Aroyl-3-arylthioureas are an exceptionally important group of thioureas which have attracted recent interest. They have been used in the synthesis of imidazole-2-thiones (Wang *et al.*, 2005) and 2-(aroylimino)-3-aryl-4-phenyl-1,3-thiazolines (Saeed *et al.*, 2008). We report here the structure of the title thiourea derivative (I), Fig. 1.

With the exception of the C12 atoms of two methyl groups of the *t*-butyl substituent all of the non-hydrogen atoms of the title compound, C_13_H_15_N_2_O_3_SCl lie on a mirror plane. Intramolecular N2—H2N···O1 and C9—H9···S1 hydrogen bonds each form a 6-membered ring, also in the mirror plane. Bond distances within the molecule are normal (Allen *et al.* 1987) and similar to those observed in comparable structures (Saeed & Flörke 2007*a*,b; Yusof *et al.* 2006, 2008). Despite the presence of both amide and thioamide groups in the molecule, no intermolecular hydrogen bonds are observed in the crystal structure. Weak π–π contacts between neighbouring C4···C9 rings, Fig 2, with centroid···centroid distances 4.2903 (17)Å and perpendicular distances between the molecular planes of 3.273Å link adjacent molecules above and below the molecular plane along *b*. These stacks of molecules form sheets parallel to the *c* axis, Fig. 3.

### Experimental

A solution of pivaloyl chloride (10 mmol) in anhydrous acetone (50 ml) was added dropwise to a suspension of potassium thiocyanate (10 mmol) in acetone (30 ml) and the reaction mixture was refluxed for 30 min. After cooling to room temperature, a solution of 2-chloro-5-nitroaniline (1.28 g, 10 mmol) in acetone (10 ml) was added and the resulting mixture refluxed for 2 h. The reaction mixture was poured into cold water and the thiourea was precipitated as a white solid. Recrystallization from ethanol gave colorless crystals of (I) (8.6 mmol, 86%). IR (KBr) cm^-1^: 3351 (free NH), 3200 (assoc. NH), 1667 (CO), 1610 (arom.), 1529 (thioureido I) 1325 II, 1160 III, 744, 762.

### Refinement

The H atoms bound to N1 and N2 and C11 were located in a difference Fourier map and their coordinates refined with *U*_iso_= 1.2*U*_eq_ (N/C). All other H atoms were refined using a riding model with d(C—H) = 0.95 Å, for aromatic H atoms with *U*_iso_= 1.2*U*_eq_ (C). For the remaining methyl groups d(C—H) = 0.98 Å, *U*_iso_ = 1.5*U*_eq_ (C).

### Crystal data

**Table:**

C_12_H_14_ClN_3_O_3_S	*F*(000) = 656
*M**_r_* = 315.77	*D*_x_ = 1.517 Mg m^−^^3^
Orthorhombic, *P**n**m**a*	Mo *K*α radiation, λ = 0.71073 Å
Hall symbol: -P 2ac 2n	Cell parameters from 2410 reflections
*a* = 9.529 (2) Å	θ = 2.3–24.6°
*b* = 6.546 (2) Å	µ = 0.44 mm^−^^1^
*c* = 22.166 (6) Å	*T* = 89 K
*V* = 1382.7 (7) Å^3^	Needle, colourless
*Z* = 4	0.36 × 0.09 × 0.06 mm

### Data collection

**Table:**

Bruker APEXII CCD area-detector diffractometer	1434 independent reflections
Radiation source: fine-focus sealed tube	1166 reflections with *I* > 2σ(*I*)
graphite	*R*_int_ = 0.069
ω scans	θ_max_ = 25.7°, θ_min_ = 1.8°
Absorption correction: multi-scan (*SADABS*; Bruker, 2006)	*h* = −11→11
*T*_min_ = 0.833, *T*_max_ = 0.974	*k* = −6→8
12525 measured reflections	*l* = −26→26

### Refinement

**Table:**

Refinement on *F*^2^	Primary atom site location: structure-invariant direct methods
Least-squares matrix: full	Secondary atom site location: difference Fourier map
*R*[*F*^2^ > 2σ(*F*^2^)] = 0.033	Hydrogen site location: inferred from neighbouring sites
*wR*(*F*^2^) = 0.092	H atoms treated by a mixture of independent and constrained refinement
*S* = 1.09	*w* = 1/[σ^2^(*F*_o_^2^) + (0.0461*P*)^2^ + 0.3426*P*] where *P* = (*F*_o_^2^ + 2*F*_c_^2^)/3
1434 reflections	(Δ/σ)_max_ < 0.001
130 parameters	Δρ_max_ = 0.27 e Å^−^^3^
0 restraints	Δρ_min_ = −0.43 e Å^−^^3^

### Special details

**Table:**

Geometry. All e.s.d.'s (except the e.s.d. in the dihedral angle between two l.s. planes) are estimated using the full covariance matrix. The cell e.s.d.'s are taken into account individually in the estimation of e.s.d.'s in distances, angles and torsion angles; correlations between e.s.d.'s in cell parameters are only used when they are defined by crystal symmetry. An approximate (isotropic) treatment of cell e.s.d.'s is used for estimating e.s.d.'s involving l.s. planes.
Refinement. Refinement of *F*^2^ against ALL reflections. The weighted *R*-factor *wR* and goodness of fit *S* are based on *F*^2^, conventional *R*-factors *R* are based on *F*, with *F* set to zero for negative *F*^2^. The threshold expression of *F*^2^ > σ(*F*^2^) is used only for calculating *R*-factors(gt) *etc*. and is not relevant to the choice of reflections for refinement. *R*-factors based on *F*^2^ are statistically about twice as large as those based on *F*, and *R*- factors based on ALL data will be even larger.

### Fractional atomic coordinates and isotropic or equivalent isotropic displacement parameters (Å^2^)

**Table:**

	*x*	*y*	*z*	*U*_iso_*/*U*_eq_	
C1	0.1946 (3)	0.2500	0.36075 (12)	0.0163 (6)	
O1	0.3187 (2)	0.2500	0.34713 (8)	0.0217 (5)	
N1	0.1523 (3)	0.2500	0.42088 (10)	0.0184 (5)	
H1N	0.070 (4)	0.2500	0.4268 (16)	0.037 (11)*	
C2	0.2314 (3)	0.2500	0.47417 (11)	0.0143 (6)	
S1	0.14289 (7)	0.2500	0.53901 (3)	0.0192 (2)	
N2	0.3713 (2)	0.2500	0.46466 (10)	0.0143 (5)	
H2N	0.397 (3)	0.2500	0.4222 (15)	0.030 (9)*	
C4	0.4846 (3)	0.2500	0.50580 (12)	0.0130 (6)	
C5	0.6210 (3)	0.2500	0.48174 (11)	0.0147 (6)	
Cl1	0.64439 (7)	0.2500	0.40389 (3)	0.0181 (2)	
C6	0.7399 (3)	0.2500	0.51801 (12)	0.0168 (6)	
H6	0.8305	0.2500	0.5002	0.020*	
C7	0.7261 (3)	0.2500	0.58025 (12)	0.0183 (6)	
H7	0.8060	0.2500	0.6059	0.022*	
C8	0.5911 (3)	0.2500	0.60368 (11)	0.0167 (6)	
N3	0.5743 (3)	0.2500	0.67027 (10)	0.0204 (5)	
O2	0.6808 (2)	0.2500	0.70124 (9)	0.0286 (5)	
O3	0.4544 (2)	0.2500	0.69074 (9)	0.0317 (6)	
C9	0.4711 (3)	0.2500	0.56877 (11)	0.0147 (6)	
H9	0.3810	0.2500	0.5871	0.018*	
C10	0.0727 (3)	0.2500	0.31561 (11)	0.0170 (6)	
C11	0.1330 (3)	0.2500	0.25185 (13)	0.0308 (8)	
H11A	0.059 (4)	0.2500	0.2261 (16)	0.037*	
H11B	0.188 (2)	0.132 (3)	0.2442 (10)	0.037*	
C12	−0.0158 (2)	0.0571 (3)	0.32488 (9)	0.0229 (5)	
H12A	−0.0897	0.0515	0.2942	0.034*	
H12B	−0.0585	0.0602	0.3651	0.034*	
H12C	0.0442	−0.0638	0.3213	0.034*	

### Atomic displacement parameters (Å^2^)

**Table:**

	*U*^11^	*U*^22^	*U*^33^	*U*^12^	*U*^13^	*U*^23^
C1	0.0149 (14)	0.0200 (15)	0.0142 (14)	0.000	−0.0001 (11)	0.000
O1	0.0135 (10)	0.0360 (12)	0.0155 (9)	0.000	0.0018 (8)	0.000
N1	0.0109 (13)	0.0317 (14)	0.0125 (11)	0.000	0.0003 (10)	0.000
C2	0.0129 (13)	0.0185 (14)	0.0113 (13)	0.000	−0.0021 (10)	0.000
S1	0.0149 (4)	0.0307 (4)	0.0121 (3)	0.000	0.0020 (3)	0.000
N2	0.0124 (12)	0.0196 (12)	0.0110 (11)	0.000	−0.0008 (9)	0.000
C4	0.0134 (13)	0.0108 (13)	0.0146 (13)	0.000	−0.0025 (10)	0.000
C5	0.0183 (14)	0.0129 (13)	0.0128 (13)	0.000	−0.0007 (11)	0.000
Cl1	0.0149 (4)	0.0262 (4)	0.0132 (3)	0.000	0.0020 (2)	0.000
C6	0.0128 (14)	0.0169 (14)	0.0209 (14)	0.000	0.0010 (11)	0.000
C7	0.0190 (15)	0.0148 (14)	0.0210 (14)	0.000	−0.0057 (12)	0.000
C8	0.0213 (15)	0.0150 (14)	0.0139 (13)	0.000	−0.0018 (11)	0.000
N3	0.0258 (14)	0.0213 (13)	0.0142 (12)	0.000	−0.0011 (10)	0.000
O2	0.0284 (12)	0.0391 (13)	0.0182 (10)	0.000	−0.0114 (9)	0.000
O3	0.0266 (12)	0.0527 (15)	0.0157 (10)	0.000	0.0031 (9)	0.000
C9	0.0165 (14)	0.0122 (13)	0.0154 (13)	0.000	−0.0002 (11)	0.000
C10	0.0147 (14)	0.0258 (15)	0.0104 (13)	0.000	−0.0018 (11)	0.000
C11	0.0193 (17)	0.062 (2)	0.0115 (14)	0.000	−0.0013 (13)	0.000
C12	0.0222 (11)	0.0220 (11)	0.0245 (10)	0.0000 (9)	−0.0078 (8)	−0.0021 (9)

### Geometric parameters (Å, °)

**Table:**

C1—O1	1.221 (3)	C7—C8	1.387 (4)
C1—N1	1.392 (3)	C7—H7	0.9500
C1—C10	1.533 (4)	C8—C9	1.380 (4)
N1—C2	1.401 (3)	C8—N3	1.485 (3)
N1—H1N	0.80 (4)	N3—O2	1.226 (3)
C2—N2	1.349 (3)	N3—O3	1.229 (3)
C2—S1	1.667 (3)	C9—H9	0.9500
N2—C4	1.414 (3)	C10—C11	1.526 (4)
N2—H2N	0.97 (3)	C10—C12	1.533 (3)
C4—C9	1.402 (3)	C10—C12^i^	1.533 (3)
C4—C5	1.405 (4)	C11—H11A	0.91 (4)
C5—C6	1.389 (4)	C11—H11B	0.95 (2)
C5—Cl1	1.740 (3)	C12—H12A	0.9800
C6—C7	1.386 (4)	C12—H12B	0.9800
C6—H6	0.9500	C12—H12C	0.9800
			
O1—C1—N1	121.1 (2)	C9—C8—C7	123.9 (2)
O1—C1—C10	124.9 (2)	C9—C8—N3	117.9 (2)
N1—C1—C10	113.9 (2)	C7—C8—N3	118.2 (2)
C1—N1—C2	130.6 (2)	O2—N3—O3	124.3 (2)
C1—N1—H1N	116 (3)	O2—N3—C8	117.9 (2)
C2—N1—H1N	113 (3)	O3—N3—C8	117.8 (2)
N2—C2—N1	113.6 (2)	C8—C9—C4	118.8 (2)
N2—C2—S1	129.4 (2)	C8—C9—H9	120.6
N1—C2—S1	117.0 (2)	C4—C9—H9	120.6
C2—N2—C4	130.8 (2)	C11—C10—C12	109.35 (14)
C2—N2—H2N	113.7 (19)	C11—C10—C12^i^	109.35 (14)
C4—N2—H2N	115.5 (19)	C12—C10—C12^i^	111.0 (2)
C9—C4—C5	117.6 (2)	C11—C10—C1	108.6 (2)
C9—C4—N2	124.9 (2)	C12—C10—C1	109.25 (14)
C5—C4—N2	117.5 (2)	C12^i^—C10—C1	109.25 (14)
C6—C5—C4	122.3 (2)	C10—C11—H11A	107 (2)
C6—C5—Cl1	118.0 (2)	C10—C11—H11B	111.9 (14)
C4—C5—Cl1	119.7 (2)	H11A—C11—H11B	108.5 (18)
C7—C6—C5	119.9 (2)	C10—C12—H12A	109.5
C7—C6—H6	120.0	C10—C12—H12B	109.5
C5—C6—H6	120.0	H12A—C12—H12B	109.5
C6—C7—C8	117.4 (2)	C10—C12—H12C	109.5
C6—C7—H7	121.3	H12A—C12—H12C	109.5
C8—C7—H7	121.3	H12B—C12—H12C	109.5
			
O1—C1—N1—C2	0.0	C6—C7—C8—N3	180.0
C10—C1—N1—C2	180.0	C9—C8—N3—O2	180.0
C1—N1—C2—N2	0.0	C7—C8—N3—O2	0.000 (1)
C1—N1—C2—S1	180.0	C9—C8—N3—O3	0.000 (1)
N1—C2—N2—C4	180.0	C7—C8—N3—O3	180.0
S1—C2—N2—C4	0.000 (1)	C7—C8—C9—C4	0.000 (1)
C2—N2—C4—C9	0.000 (1)	N3—C8—C9—C4	180.0
C2—N2—C4—C5	180.0	C5—C4—C9—C8	0.000 (1)
C9—C4—C5—C6	0.0	N2—C4—C9—C8	180.0
N2—C4—C5—C6	180.0	O1—C1—C10—C11	0.0
C9—C4—C5—Cl1	180.0	N1—C1—C10—C11	180.0
N2—C4—C5—Cl1	0.0	O1—C1—C10—C12	119.21 (15)
C4—C5—C6—C7	0.000 (1)	N1—C1—C10—C12	−60.79 (15)
Cl1—C5—C6—C7	180.0	O1—C1—C10—C12^i^	−119.21 (15)
C5—C6—C7—C8	0.000 (1)	N1—C1—C10—C12^i^	60.79 (15)
C6—C7—C8—C9	0.000 (1)		

Symmetry codes: (i) *x*, −*y*+1/2, *z*.

### Hydrogen-bond geometry (Å, °)

**Table:**

*D*—H···*A*	*D*—H	H···*A*	*D*···*A*	*D*—H···*A*
C9—H9···S1	0.95	2.51	3.197 (3)	130
N2—H2N···O1	0.97 (3)	1.82 (3)	2.653 (3)	141 (3)
